# Implementation of State Vaccine Incentive Lottery Programs and Uptake of COVID-19 Vaccinations in the United States

**DOI:** 10.1001/jamanetworkopen.2021.38238

**Published:** 2021-12-09

**Authors:** Binod Acharya, Chandra Dhakal

**Affiliations:** 1 Urban Health Collaborative, Drexel University, Philadelphia, Pennsylvania; 2 Department of Agricultural and Applied Economics, University of Georgia, Athens

## Abstract

**Importance:**

There is limited evidence on whether COVID-19 vaccine incentive lotteries help to increase vaccine uptake.

**Objective:**

To estimate whether and to what extent statewide implementation of lottery programs is associated with COVID-19 vaccine uptake.

**Design, Setting, and Participants:**

This cross-sectional study used data from 403 714 adults who answered the question, “have you received a COVID-19 vaccine?” on the Household Pulse Survey (HPS) and state-level daily vaccination rates for 11 states implementing a vaccine lottery program (ie, treated states) and 28 states with no such program (ie, control states). A difference-in-difference (DiD) analysis used responses to the HPS question and compared changes in vaccine uptake between treated and control states. The augmented synthetic control (ASC) analysis compared state-level daily new vaccination rates in treated states with their synthetic versions constructed from a donor pool of control states. Data were analyzed from March 17 to July 5, 2021.

**Exposures:**

Announcement of the vaccine lottery programs and states implementing such programs.

**Main Outcomes and Measures:**

The outcome variable in the DiD analysis was the binary indicator of self-reported vaccination status. In the ASC analysis, it was the state-level daily new first vaccine dose administered per 100 000 people (logarithm scale).

**Results:**

A total of 403 714 individuals (mean [SD] age, 52.7 [15.7] years; 239 563 [weighted percentage, 51.6%] women; 31 746 [weighted percentage, 11.9%] Black; 39 709 [weighted percentage, 18.2%] Hispanic; 334 034 [weighted percentage, 76.4%] White) responded to the question in HPS regarding vaccination status. Overall, 80 949 respondents (weighted percentage, 28.1%) in the HPS were unvaccinated. A pooled analysis from both methods indicated that the lottery programs were associated with increased vaccinations. The ASC analysis revealed that lottery programs were associated with an increase of 0.208 log points (95% CI, 0.004-0.412 log points), implying an average 23.12% increment in the new daily vaccination rate. In state-specific analyses, both methods suggested that the vaccine lottery programs were helpful in Ohio (0.09 log points; *P* < .001), Maryland (0.26 log points; *P* < .001), Oregon (0.15 log points; *P* = .002), and Washington (0.37 log points; *P* < .001) but not in Arkansas, Kentucky, and West Virginia. The ASC analysis found that lottery programs were positively associated with vaccinations in New Mexico (0.32 log points; *P* < .001) and New York (0.33 log points; *P* = .001).

**Conclusions and Relevance:**

The findings of this study suggest that lottery programs may be associated with decreased COVID-19 vaccine hesitancy, but that success might differ across states. The findings could also be useful in the ongoing debate on persuading US residents who are not yet vaccinated.

## Key Points

**Question:**

Have vaccine lottery programs in the United States been associated with increases in the COVID-19 vaccination rate?

**Findings:**

This cross-sectional study of 403 714 adult participants in the Household Pulse Survey and state daily vaccination rates found that vaccine lottery programs were associated with an increase in COVID-19 vaccination rates overall and in some states but not in others.

**Meaning:**

These findings suggest that policy makers should take the heterogeneous response to vaccine incentive lottery programs into account when implementing similar policies.

## Teaser

This cross-sectional study estimates whether statewide implementation of vaccine incentive lottery programs is associated with COVID-19 vaccine uptake.

## Introduction

COVID-19 vaccines administered in the United States are safe and effective at lowering COVID-19 incidence, death, and hospitalization rates.^1^ The federal and local governments have put forward various strategies to increase the vaccination rate, including making the vaccine available to the public free of cost. Nevertheless, the vaccination rate in the US is less than the suggested threshold for herd immunity^2,3^ and is falling. Many states have launched incentive programs to boost vaccination, including vaccine lotteries in which a vaccinated individual is eligible to win a big cash prize.

Previous studies on the association between vaccine incentive programs and vaccine uptake have found mixed results. Cash incentives might help people change their behavioral health decision-making,^4,5^ while other researchers argue that external incentives could weaken the intrinsic motivation to take the desired action.^6^ Some maintain that monetary incentives, particularly the big cash prizes, may raise doubts about the safety of the vaccine and perpetuate vaccine skepticism^7^ and are ethically questionable.^8^

Ohio announced its lottery program, “Vax-a-Million,” on May 13, 2021, and after the initial media reports of its success, many other states adopted similar programs.^9^ However, there remains considerable uncertainty about whether such programs are successful. An interrupted time-series analysis of daily vaccinations in Ohio did not find a meaningful association of lottery incentives with vaccine uptake.^10^ Two recent reports on Ohio’s vaccine lottery program^11,12^ differ in their conclusion. The available studies are limited to a single state, and the results may not be generalizable to other states, which likely differ in key contextual factors related to public health.

In this article, we use 2 methodological approaches and 2 sources of data to investigate whether lottery-based incentives are associated with an increase in vaccination rates across multiple states in the United States. To our knowledge, this is the first study of its kind evaluating the association of this policy with vaccination rates in multiple states using both the individual responses of vaccination status and state-level daily vaccination data.

## Methods

The data set for this cross-sectional study was deidentified and publicly available. The institutional review board at the University of Georgia determined that it was not human participant research and therefore waived the requirement for informed consent. This study followed the Strengthening the Reporting of Observational Studies in Epidemiology (STROBE) reporting guideline.

### Study Population and Data

We conducted a difference-in-difference (DiD) analysis using data from the Household Pulse Survey (HPS), an online, nationally representative survey (average response rate, 7%) conducted by the US Census Bureau and aimed at understanding the impacts of the COVID-19 pandemic on American households.^13^ The HPS assigns a person-weight to each survey respondent to make the sample representative of the entire US population. We used the microdata with respondents aged 18 and older from survey weeks 27 to 33, corresponding to March 17 to July 5, 2021. The outcome of interest was a binary indicator of individual vaccination status based on the question, “Have you received a COVID-19 vaccine?” We controlled for individual and household characteristics, such as age, gender, race and ethnicity, marital status, household size, presence of children, homeownership status, employment status, supplemental nutrition assistance program (SNAP) recipient status, social security benefits recipient status, unemployment insurance recipient status, educational attainment, and household income. The HPS categorized ethnicity as Hispanic and non-Hispanic and race as Asian alone, Black alone, White alone, and other. We collapsed Asian alone and other in our model because of smaller samples in these 2 groups. Given that Ohio^14^ ended its vaccine lottery program on June 22, 2021, we limited the timeframe to the program’s end date for Ohio.

In the augmented synthetic control (ASC) analysis, we used state-level data of daily first dose of vaccine administration combined with state-level time-invariant covariates. We obtained the daily first dose of COVID-19 vaccination rate per 100 000 people (7-day moving average, natural logarithm scale) for March 17 to July 5 from Opportunity Insight.^15^ The study window corresponds to the timeframe of the HPS. We used state-level covariates, such as demographic characteristics (proportion aged ≤14 years, proportion aged ≥65 years, proportion Hispanic residents, and proportion Black residents) and socioeconomic characteristics (proportion of the population with a Bachelor’s degree or greater, proportion of people in the labor force, median household income, unemployment rate, and proportion with private health insurance) from American Community Survey 2019 annual estimates; partisan leaning index from FiveThirtyEight^16^; and geographic variables (latitude, longitude, and census division) and public-health related characteristics (prepandemic life expectancy at birth) from the US Centers for Disease Control and Prevention. Many of these covariates are associated with vaccine hesitancy^17^ and are typically used in public health literature.

The following 11 states that announced the vaccine lottery programs with prizes of at least $1 million before June 10, 2021, were considered as the treated states: Arkansas, California, Colorado, Kentucky, Maryland, Ohio, Oregon, New Mexico, New York, Washington, and West Virginia (eTable 1 in the Supplement). The analysis was performed for each state separately and by pooling them together. Illinois and Delaware were excluded because they had other major incentives targeted to increase vaccination rates.

### Statistical Analysis

First, we estimated the association between the lottery incentive programs and vaccinations with the DiD approach using linear regression models with fixed effects. The DiD analysis is extensively practiced in policy evaluation; in DiD analyses, a change in outcome in the treated unit(s) in the posttreatment period is compared with the control unit(s).^18,19,20^ This approach assumes a similar trend between treated and control units in the absence of treatment. We fitted separate models for each treated state and a model for pooled analysis. In our models, the outcome variable was the binary indicator of vaccination status. The interaction term between treatment status (a dummy labeled *Treat*) and postintervention period (a dummy labeled *Post*) is the primary variable of interest. The coefficient of the interaction term (*Treat* × *Post*) measures the proportional change in vaccine uptake associated with the vaccine lottery program implementation. We also included state-level fixed effects to control the fixed differences across states and survey week–level fixed effects to absorb the temporal variation in vaccine uptake.

We applied dynamic event study parameterization to partially test parallel trend assumptions and calculate the lead and lag estimates.^21^ Standard errors were clustered at the state level. To calculate the pooled estimator, combining all treated states, we applied the method by Sun and Abraham.^22^ The method builds on a growing body of literature on DiD with staggered adoption^23,24,25^ and deals with scenarios in which treatment is dynamic and treatment effects are heterogeneous across treated units. The DiD analysis was carried out using Stata version 17 (StataCorp).

Next, we evaluated the association between vaccine lottery programs and vaccination rates with an ASC analysis. The synthetic control method (SCM) is popular in policy evaluations that estimate the counterfactual outcome.^26^ It addresses the arbitrariness in selecting controls in traditional matching methods by explicitly choosing a data-driven approach.^27,28,29^ The SCM method works by creating a synthetic version of each treated state by a weighted combination of control states in the donor pool. The pretreatment outcome of treated states resembles their synthetic counterparts. Building on the SCM, an ASC method has been proposed^30^ that provides a way to debias the original SCM estimates when a pretreatment fit between a treated and synthetic unit is imperfect. For details on the ASC method, please refer to Ben-Michael et al.^30^

Because of the high variability in the outcome variable, we modeled the natural log of the daily vaccination rate. We specified the ridge-regularized linear regression as the outcome model and allowed nonnegative weights to improve pretreatment fit while minimizing extrapolation outside the convex hull.^30^ The ASC estimator would still be the linear combination of the control units, and the regularization parameter directly controls the level of extrapolation by penalizing the distance from SCM weights. The regularization parameter is selected via cross-validation.^30^ We applied synthetic control with a staggered adoption framework^31^ to calculate the combined, pooled results in all treated states. This approach accounts for the variation in treatment timing in different treated states. The ASC analysis was carried out using R version 4.0.3 (R Project for Statistical Computing) using the augsynth package.^32^ Statistical significance was *P* < .05, and all tests were 2-tailed.

Because of the gradual vaccine rollout programs, a substantial proportion of the population was already eligible to receive vaccines before they became available to the general public. We also anticipated that the enthusiasm brought about by the announcement of lottery programs might fade away over time. We performed sensitivity analyses to assess whether our ASC estimates were robust to change in the sampling window by limiting the time frame from when the general public became eligible for vaccination to 2 weeks after the program announcement.

## Results

Table 1 describes the characteristics of the population from the HPS in treated and control states. DiD analysis used 403 714 individuals (mean [SD] age, 52.7 [15.7] years; 239 563 [weighted percentage, 51.6%] women; 31 746 [weighted percentage, 11.9%] Black; 39 709 [weighted percentage, 18.2%] Hispanic; 334 034 [weighted percentage, 76.4%] White). Overall, 80 949 (weighted percentage, 28.1%) were unvaccinated, and 322 765 (weighted percentage, 71.9%) were vaccinated. The 2667 individuals who did not answer the question of interest were dropped. All percentages incorporate the sampling weight provided by the US Census Bureau. The mean (SD) age in the vaccinated group was higher compared with the unvaccinated group (54.5 [15.5] years vs 45.6 [14.3] years; *P* < .001). The unvaccinated group had a lower percentage of women (49 667 [50.6%] vs 189 896 [52.0%]; *P* < .001) and a higher percentage of Black individuals (8257 [14.7%] vs 23 489 [10.8%]; *P* < .001), Hispanic individuals (9758 [20.3%] vs 29 951 [17.4%]; *P* < .001), SNAP participants (9670 [15.2%] vs 15 547 [7.4%]; *P* < .001), and unemployment insurance recipients (10 213 [15.1%] vs 22 382 [9.0%]; *P* < .001) relative to vaccinated group. The unvaccinated group, on average, had a lower household income and education status compared with the vaccinated group. The trend of the daily state-specific first dose of COVID-19 vaccination rate per 100 000 (7-day moving average) from March 17 through July 5, 2021, appeared to decrease after plateauing in in mid-April (eFigure 1 in the Supplement).

**Table 1.** Demographic and Socioeconomic Characteristics of the Study Sample From the Household Pulse Survey, 2021

Characteristics	No. (weighted %)			*P* value^a^
	Full sample (N = 403 714; weighted N = 1 423 283 582)	Unvaccinated (n = 80 949; weighted n = 399 236 069)	Vaccinated (n = 322 765; weighted n = 1 024 047 512)	
Age, mean (SD), y	52.7 (15.7)	45.6 (14.3)	54.5 (15.5)	<.001
Female	239 563 (51.6)	49 667 (50.6)	189 896 (52.0)	<.001
Male	164 151 (48.4)	31 282 (49.4)	132 869 (48.0)	
Race				
White alone	334 034 (76.4)	64 667 (73.6)	269 367 (77.5)	<.001
Black alone	31 746 (11.9)	8257 (14.7)	23 489 (10.8)	
Asian alone	20 555 (5.9)	2713 (3.6)	17 842 (6.3)	
Other races^b^	17 379 (5.8)	5312 (8.1)	12 067 (4.9)	
Hispanic	39 709 (18.2)	9758 (20.3)	29 951 (17.4)	<.001
Married	236 923 (55.1)	42 034 (46.3)	194 889 (58.5)	<.001
Unemployed	163 167 (41.1)	30 013 (39.9)	133 154 (41.5)	<.001
SNAP participant	25 217 (9.6)	9670 (15.2)	15 547 (7.4)	<.001
UI recipient	32 595 (10.7)	10 213 (15.1)	22 382 (9.0)	<.001
SSA recipient	124 903 (25.6)	14 211 (14.8)	110 692 (29.9)	<.001
Education				
<High school	8394 (8.4)	8394 (12.4)	8394 (6.9)	<.001
High school and some college degree	131 185 (51.4)	131 185 (61.0)	131 185 (47.7)	
College degree	264 135 (40.2)	264 135 (26.5)	264 135 (45.5)	
Household income range, $				
<25 000	30 375 (14.4)	30 375 (21.8)	30 375 (11.7)	<.001
25 000-34 999	25 565 (11.4)	25 565 (13.9)	25 565 (10.6)	
35 000-49 999	31 302 (12.2)	31 302 (14.0)	31 302 (11.6)	
50 000-74 999	51 799 (17.8)	51 799 (18.0)	51 799 (17.8)	
75 000-99 999	43 409 (13.3)	43 409 (11.8)	43 409 (13.9)	
100 000-149 999	55 806 (15.6)	55 806 (11.5)	55 806 (17.0)	
150 000-199 999	27 746 (7.2)	27 746 (4.5)	27 746 (8.2)	
≥200 000	33 896 (8.1)	33 896 (4.5)	33 896 (9.3)	
Survey wave				
March 17-29	63 079 (14.6)	63 079 (27.7)	63 079 (9.5)	<.001
April 14-26	55 842 (14.7)	55 842 (16.1)	55 842 (14.1)	
April 28-May 10	63 860 (14.7)	63 860 (13.4)	63 860 (15.2)	
May 12-24	59 433 (14.7)	59 433 (12.1)	59 433 (15.7)	
May 26-June 7	57 873 (14.7)	57 873 (11.0)	57 873 (16.1)	
June 9-21	55 746 (14.7)	55 746 (11.1)	55 746 (16.1)	
June 23-July 5	47 881 (12.0)	47 881 (8.7)	47 881 (13.3)	

Abbreviations: SNAP, supplemental nutrition assistance program; SSA, Social Security Administration; UI, unemployment insurance.

^a^ The *t *test was used to compare mean age, and the χ^2^ test was used in all other variables.

^b^ Includes multiracial and unknown race.

**Figure 1.**
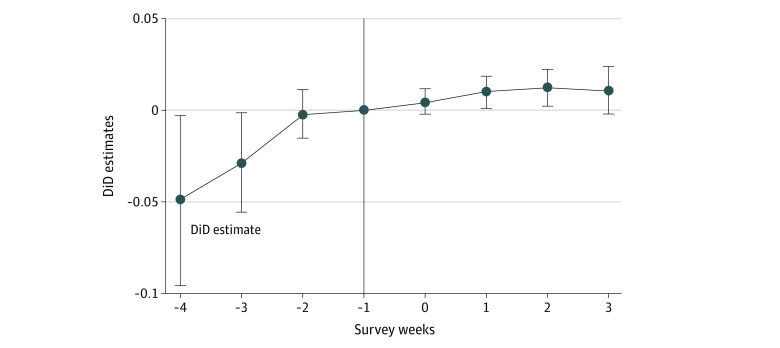
Difference-in-Difference (DiD) Estimates From Dynamic Event Study With Staggered Adoption for Pooled Analysis On the x-axis, 0 indicates the week the incentive started.

In the pooled DiD analysis, we first evaluated the validity of the parallel trend assumption using the dynamic event study approach.^22^ Figure 1 displays the event study analysis results, in which 1 survey wave before the program announcement was considered a reference week. We found no evidence of a differential trend of vaccinations between treated and control states.

Table 2 presents estimates from the DiD approach. The coefficient of *Treat* × *Post* in the pooled analysis column shows the association between lottery program and vaccination from the pooled analysis, considering variation in treatment timing. The result showed that the lottery programs were significantly associated with a 2.1% (95% CI: 0.7%-3.5%; *P* < .001) increase in vaccine uptake, controlling for observables. Table 2 also provides DiD estimates for each of the treated states. California (2.5%; 95% CI: 1.5%-3.5%; *P* < .001), Colorado (2.2%; 95% CI: 1.2%-3.2%; *P* < .001), Maryland (6.4%; 95% CI: 5.4%-7.4%; *P* < .001), Ohio (5.4%; 95% CI: 4.4%-6.4%; *P* < .001), Oregon (6.6%; 95% CI: 5.6%-7.6%; *P* < .001), and Washington (3.8%; 95% CI: 2.8%-4.8%; *P* < .001) showed a positive and significant association between the lottery program and vaccinations. The magnitude of the association was largest in Oregon, followed by Maryland and Ohio. In contrast, the lottery programs were not associated with vaccinations in New Mexico and New York. DiD estimates for Arkansas, Kentucky, and West Virginia implied the opposite association between the lottery program and vaccine uptake. However, state-specific results could be imprecise and need caution in interpretation given that the event study graphs provide little evidence of parallel pre-trend assumption (eFigure 2 in the Supplement). Among the covariates used for adjustment in the DiD analysis, having a higher education, being a woman, owning a house, and receiving social security benefits were positively associated with the vaccine uptake, while being unemployed, participating in SNAP, and having children was negatively associated.

**Table 2.** Association Between Vaccine Lottery Program and Vaccine Uptake From the Difference-in-Difference Analysis Using the Household Pulse Survey, 2021

Variable	Estimate (SE)											
	Pooled analysis (N =301 080)	State-specific analysis										
		AR (n = 200 327)	CA (n = 196 174)	CO (n = 205 720)	KY (n = 200 383)	MD (n = 205 433)	NM (n = 203 836)	NY (n = 203 401)	OH (n = 175 621)	OR (n = 205 836)	WA (n = 210 807)	WV (n = 199 696)
Post × Treat	0.021 (0.007)^a^	−0.012 (0.005)^b^	0.025 (0.005)^a^	0.022 (0.005)^a^	−0.045 (0.005)^a^	0.064 (0.005)^a^	−0.002 (0.005)	−0.002 (0.005)	0.054 (0.005)^a^	0.066 (0.005)^a^	0.038 (0.005)^a^	−0.045 (0.005)^a^
Age, y	0.005 (0)^a^	0.005 (0)^a^	0.005 (0)^a^	0.005 (0)^a^	0.005 (0)^a^	0.005 (0)^a^	0.005 (0)^a^	0.005 (0)^a^	0.005 (0)^a^	0.005 (0)^a^	0.005 (0)^a^	0.005 (0)^a^
Female	0.014 (0.004)^a^	0.013 (0.006)	0.014 (0.005)^a^	0.014 (0.006)^b^	0.013 (0.006)^b^	0.013 (0.005)^b^	0.013 (0.006)^b^	0.012 (0.005)^b^	0.015 (0.006)^b^	0.013 (0.006)^b^	0.012 (0.005)^b^	0.013 (0.006)^b^
Married	0.012 (0.003)^a^	0.011b (0.005)^b^	0.014 (0.005)^a^	0.012 (0.005)^b^	0.011b (0.005)^b^	0.013 (0.005)^b^	0.012 (0.005)^b^	0.010 (0.004)^b^	0.012 (0.005)^b^	0.011 (0.005)^b^	0.011 (0.005)^b^	0.011 (0.005)^b^
Household size, No.	−0.013 (0.002)^a^	−0.013 (0.003)^a^	−0.014 (0.003)^a^	−0.014 (0.003)^a^	−0.014 (0.003)^a^	−0.013 (0.003)^a^	−0.013 (0.003)^a^	−0.013 (0.003)^a^	−0.013 (0.003)^a^	−0.014 (0.003)^a^	−0.013 (0.003)^a^	−0.013 (0.003)^a^
Children	−0.051 (0.007)^a^	−0.060 (0.005)^a^	−0.045 (0.01)^a^	−0.058 (0.005)^a^	−0.058 (0.005)^a^	−0.058 (0.005)^a^	−0.058 (0.006)^a^	−0.058 (0.005)^a^	−0.058 (0.007)^a^	−0.058 (0.006)^a^	−0.058 (0.005)^a^	−0.058 (0.006)^a^
Home ownership	0.030 (0.005)^a^	0.031 (0.007)^a^	0.027 (0.005)^a^	0.032 (0.007)^a^	0.030 (0.007)^a^	0.031 (0.007)^a^	0.031 (0.007)^a^	0.029 (0.007)^a^	0.030 (0.007)^a^	0.030 (0.007)^a^	0.031 (0.007)^a^	0.030 (0.007)^a^
Unemployed	−0.032 (0.008)^a^	−0.021 (0.006)^a^	−0.029b (0.011)^b^	−0.020 (0.006)^a^	−0.022 (0.006)^a^	−0.021 (0.006)^a^	−0.020 (0.006)^a^	−0.023 (0.006)^a^	−0.021 (0.007)^a^	−0.021 (0.006)^a^	−0.020 (0.006)^a^	−0.021 (0.006)^a^
SNAP participant	−0.068 (0.005)^a^	−0.064 (0.006)^a^	−0.072 (0.006)^a^	−0.064 (0.006)^a^	−0.065 (0.006)^a^	−0.061 (0.006)^a^	−0.062 (0.006)^a^	−0.067 (0.006)^a^	−0.071 (0.007)^a^	−0.063 (0.005)^a^	−0.064 (0.005)^a^	−0.064 (0.006)^a^
UI recipient	−0.024 (0.006)^a^	−0.032 (0.007)^a^	−0.024 (0.008)^a^	−0.034 (0.007)^a^	−0.032 (0.007)^a^	−0.032 (0.007)^a^	−0.032 (0.007)^a^	−0.029 (0.007)^a^	−0.036 (0.007)^a^	−0.033 (0.007)^a^	−0.034 (0.007)^a^	−0.032 (0.007)^a^
SSA recipient	0.066 (0.006)^a^	0.062 (0.008)^a^	0.066 (0.009)^a^	0.061 (0.008)^a^	0.062 (0.008)^a^	0.060 (0.008)^a^	0.059 (0.008)^a^	0.063 (0.008)^a^	0.065 (0.009)^a^	0.061 (0.008)^a^	0.061 (0.008)^a^	0.061 (0.008)^a^
Race												
White alone	[Reference]	[Reference]	[Reference]	[Reference]	[Reference]	[Reference]	[Reference]	[Reference]	[Reference]	[Reference]	[Reference]	[Reference]
Hispanic	0.063 (0.01)^a^	0.067 (0.015)^a^	0.065 (0.01)^a^	0.066 (0.015)^a^	0.068 (0.015)^a^	0.066 (0.015)^a^	0.066 (0.015)^a^	0.060 (0.016)^a^	0.066 (0.015)^a^	0.066 (0.015)^a^	0.066 (0.015)^a^	0.067 (0.016)^a^
Black alone	−0.011 (0.01)	−0.002 (0.01)	−0.009 (0.013)	−0.003 (0.01)	−0.001 (0.01)	−0.003 (0.009)	−0.004 (0.01)	−0.010 (0.011)	−0.001 (0.011)	−0.004 (0.01)	−0.002 (0.01)	−0.003 (0.01)
Asian or other race	0.013 (0.007)^c^	−0.001 (0.009)	0.002 (0.006)	−0.001 (0.009)	0 (0.009)	0.002 (0.009)	0.001 (0.009)	0.009 (0.012)	0.001 (0.009)	−0.001 (0.009)	0.004 (0.01)	−0.000 (0.009)
Education												
<High school	[Reference]	[Reference]	[Reference]	[Reference]	[Reference]	[Reference]	[Reference]	[Reference]	[Reference]	[Reference]	[Reference]	[Reference]
High school degree	0.035 (0.013)^b^	0.046 (0.012)^a^	0.027 (0.016)^c^	0.045 (0.012)^a^	0.045 (0.013)^a^	0.047 (0.013)^a^	0.046 (0.013)^a^	0.055 (0.014)^a^	0.043 (0.012)^a^	0.045 (0.013)^a^	0.045 (0.012)^a^	0.047 (0.013)^a^
Some college degree	0.095 (0.013)^a^	0.100 (0.014)^a^	0.086 (0.014)^a^	0.100 (0.014)^a^	0.100 (0.014)^a^	0.101 (0.014)^a^	0.100 (0.014)^a^	0.107 (0.016)^a^	0.104 (0.015)^a^	0.100 (0.014)^a^	0.100 (0.014)^a^	0.101 (0.015)^a^
College degree	0.187 (0.015)^a^	0.202 (0.017)^a^	0.184 (0.018)^a^	0.200 (0.016)^a^	0.201 (0.017)^a^	0.201 (0.017)^a^	0.201 (0.017)^a^	0.205 (0.016)^a^	0.207 (0.016)^a^	0.201 (0.017)^a^	0.200 (0.016)^a^	0.202 (0.017)^a^
Household income (in $10 000)	0.005 (0.001)^a^	0.006 (0.001)^a^	0.005 (0.001)^a^	0.006 (0.001)^a^	0.006 (0.001)^a^	0.006 (0.001)^a^	0.006 (0.001)^a^	0.006 (0.001)^a^	0.006 (0.001)^a^	0.006 (0.001)^a^	0.006 (0.001)^a^	0.006 (0.001)^a^
Survey waves												
March 17-29	[Reference]	[Reference]	[Reference]	[Reference]	[Reference]	[Reference]	[Reference]	[Reference]	[Reference]	[Reference]	[Reference]	[Reference]
April 14-26	0.225 (0.008)^a^	0.219 (0.006)^a^	0.227 (0.008)^a^	0.224 (0.007)^a^	0.218 (0.007)^a^	0.224 (0.007)^a^	0.221 (0.006)^a^	0.217 (0.006)^a^	0.218 (0.006)^a^	0.221 (0.006)^a^	0.223 (0.006)^a^	0.220 (0.006)^a^
April 28-May 10	0.273 (0.008)^a^	0.269 (0.01)^a^	0.274 (0.008)^a^	0.275 (0.01)^a^	0.268 (0.01)^a^	0.273 (0.01)^a^	0.271 (0.01)^a^	0.269 (0.009)^a^	0.266 (0.01)^a^	0.272 (0.01)^a^	0.273 (0.009)^a^	0.269 (0.01)^a^
May 12-24	0.294 (0.009)^a^	0.285 (0.008)^a^	0.296 (0.01)^a^	0.291 (0.008)^a^	0.284 (0.008)^a^	0.289 (0.008)^a^	0.287 (0.008)^a^	0.288 (0.007)^a^	0.283 (0.009)^a^	0.287 (0.008)^a^	0.288 (0.007)^a^	0.286 (0.008)^a^
May 26-June 7	0.309 (0.009)^a^	0.305 (0.009)^a^	0.311 (0.009)^a^	0.310 (0.009)^a^	0.304 (0.01)^a^	0.310 (0.009)^a^	0.307 (0.009)^a^	0.305 (0.009)^a^	0.303 (0.01)^a^	0.308 ^a^(0.009)	0.309 (0.009)^a^	0.307 (0.009)^a^
June 9-21	0.310 (0.009)^a^	0.302 (0.009)^a^	0.309 (0.01)^a^	0.305 (0.009)^a^	0.301 (0.009)^a^	0.305 (0.009)^a^	0.303 (0.009)^a^	0.299 (0.01)^a^	0.303 (0.009)^a^	0.304 (0.009)^a^	0.305 (0.009)^a^	0.302 (0.009)^a^
June 23-July 5	0.322 (0.01)^a^	0.320 (0.011)^a^	0 (0)	0.324 (0.011)^a^	0.320 (0.011)^a^	0.322 (0.011)^a^	0.321 (0.011)^a^	0.319 (0.01)^a^	0 (0)	0.322 (0.011)^a^	0.322 (0.011)^a^	0.319 (0.011)^a^
Constant	0.137 (0.020)^a^	0.099 (0.015)^a^	0.127 (0.026)^a^	0.104 (0.015)^a^	0.103 (0.015)^a^	0.099 (0.015)^a^	0.103 (0.015)^a^	0.108 (0.013)^a^	0.080 (0.016)^a^	0.103 (0.015)^a^	0.104 (0.014)^a^	0.102 (0.015)^a^
*R* ^2^	0.193	0.190	0.193	0.191	0.189	0.192	0.189	0.191	0.199	0.191	0.190	0.189

Abbreviations: SNAP, supplemental nutrition assistance program; SSA, Social Security Administration; UI, unemployment insurance.

^a^ *P* < .01.

^b^ *P* < .05.

^c^ *P* < .10.

Summary statistics (mean and SD) of outcome and covariates used in the ASC in treated states and the donor pool of control states are presented in eTable 2 in the Supplement. In general, covariates were comparable, but the treated states had a higher vaccination rate than control states. Each donor states’ relative contributions (ie, weights) to the synthetic version of each treated state are available in eTable 3 in the Supplement. Using the ASC method, we extrapolated outside the convex hull, and thus, the weights could be negative. The weight matrix for the pooled analysis is not shown but is available on request.

**Figure 2.**
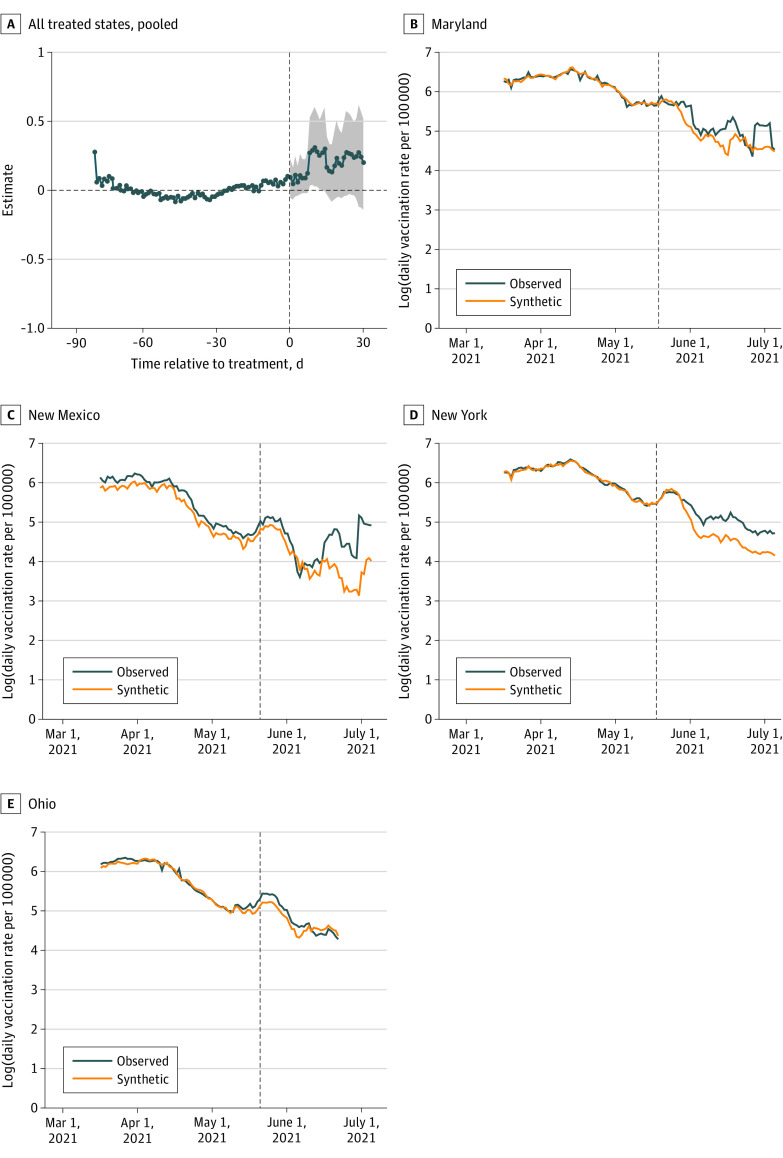
Gap Plots From Pooled ASC Analysis and Trends of Daily Vaccination Rate in Treated States and Their Synthetic Controls A, The shaded region is a pointwise 95% confidence band computed with wild bootstrap.^31,33^ B-E, The vertical reference line indicates the date of the lottery program announcement.

Figure 2A shows the estimated relative change in the daily new vaccination rate per 100 000 (log scale) between treated and synthetic states from the pooled analysis, which accounts for the staggered adoption (variation in the treatment timing across states) in multiple states.^31,33^ We found that the combined outcome of all treated states was associated with an improvement in the daily vaccination rate by 0.208 log points (95% CI, 0.004-0.412 log points). This implied an average 23.12% increment in the daily vaccination rate after the program implementation. Given that the average vaccination rate of treated states on the day of the announcement was 230 per 100 000, a 23.12% increase in the daily new vaccination rate corresponds to approximately 53 additional new vaccine doses per 100 000 people.

In Figure 2B to E and eFigure 3 in the Supplement, we plot the trajectories of daily vaccination in each treated state and its synthetic control. The pretreatment trends of vaccination rates in the treated states closely tracked those in the synthetic control, barring a few exceptions, suggesting that synthetics are generally credible counterfactuals. In the postintervention period, the vaccination in most treated states was higher than their corresponding synthetic controls.

We present gap plots in eFigure 4 in the Supplement for each of the treated states. The corresponding average treatment effects on treated (ATT) and *P* values are presented in Table 3. A substantial improvement in vaccination rate is evident in New Mexico (0.32 log points; *P* < .001), New York (0.33 log points; *P* = .001), Washington (0.37 log points; *P* < .001), Ohio (0.09 log points; *P* < .001), Oregon (0.15 log points; *P* = .002), and Maryland (0.26 log points; *P* < .001). These translate to 108 000 additional new vaccine doses in New Mexico, 744 000 in New York, 178 000 in Washington, 61 000 in Ohio, 77 000 in Oregon, and 221 000 in Maryland. However, ASC analysis did not find a positive association between lottery programs and vaccination rates in Arkansas, Kentucky, California, Colorado, and West Virginia. In Ohio, where the prize was $5 million, the marginal cost per induced vaccination was $82.

**Table 3.** ATT Estimates From the Ridge Augmented Synthetic Control Analysis

State	ATT, log points	*P* value
Arkansas	−0.062	.08
California	0.075	.63
Colorado	0.110	.63
Kentucky	−0.078	.67
Maryland	0.256	<.001
New Mexico	0.322	<.001
New York	0.331	.001
Ohio	0.093	<.001
Oregon	0.147	.002
Washington	0.366	<.001
West Virginia	0.035	.40
Overall, mean (SE)	0.208 (0.104)	NA^a^

Abbreviations: ATT, average treatment effect on treated; NA, not applicable.

^a^ In the overall ATT estimate, obtaining a *P* value on synthetic control with a staggered adoption framework is a matter of ongoing inquiry. The statistical software (multisynth function in the R package augsynth) we used does not report it. Based on the 95% confidence interval (0.004-0.412), the overall (pooled) ATT estimate is statistically significant at a 5% level.

The estimates in the sensitivity analysis were largely consistent with our results. A gap plot from the pooled analysis is available in eFigure 5 in the Supplement. From state-specific analyses, the observed vs synthetic plots and gap plots of daily vaccination rates are available in eFigure 6 and 7 in the Supplement, respectively.

## Discussion

Past work has documented that vaccine hesitancy is a global matter of concern.^34,35,36,37^ The US federal and state governments have launched programs to address misinformation about the safety and effectiveness of the COVID-19 vaccine and adopted incentive programs from free beer to lottery prizes.^38^ Little empirical evidence exists on the role of lottery incentives in improving vaccination. Our study used sophisticated policy evaluation methods to address that information bottleneck.

The DiD estimate from the pooled analysis suggests that the lottery program was associated with a 2.1% increase in vaccine uptake. When analyzed separately by state, the results were mixed: lottery programs appeared successful in most states, but not in all states. The DiD results, however, need to be interpreted with caution when the treated and control states do not provide evidence of no differential trend during the preintervention period. We also acknowledge that the selection on unobservables could still be an issue in the DiD setting. After the announcement of lottery incentives, the decision to get vaccinated may be informed differentially by unobservable across control and treated units.^39^

Consistent with the pooled DiD estimate, our pooled ASC analysis revealed a positive association between lottery programs and daily vaccination rates. Given that the adult population in 11 treated states is approximately 85 million,^40^ a 2.1% estimate from the DiD result implies that the lottery programs may have expanded the vaccine coverage to approximately 1.78 million additional adults. Similarly, our ASC estimate of 53 doses per day per 100 000 people translates into approximately 1.67 million additional adults getting vaccinated for an average of 37 posttreatment days in 11 treated states (calculated as 85 000 000 × 53 × 37/100 000). The results were mixed in the state-specific ASC analyses, with the highest ATT being observed in Washington. Among the states where vaccine uptake was positively associated with lottery programs, we estimate the number of new additional vaccinations to be approximately 108 000, 744 000, 178 000, 61 000, 77 000, and 221 000 for New Mexico, New York, Washington, Ohio, Oregon, and Maryland, respectively. The $5 million lottery program in Ohio ended within the study window, which produced the marginal cost per induced vaccination to be $82.

We recognize that vaccine acceptance may be a much more complex process with political, psychological, cultural, geographical, or socioeconomic elements involved^41,42,43,44,45^ that could explain the differential results of lottery programs in different states. In particular, we note that the states where vaccine lottery incentives did not succeed—West Virginia, Arkansas, and Kentucky—are heavily Republican,^16^ the political group less responsive to financial incentives for COVID-19 vaccinations.^17^ Future research examining vaccinations with politico-economic characteristics, the access and time to go to the nearest vaccine center, and the nature of public-health mobilization implemented by local governments may shed light on this differential response.

Our Ohio lottery program results differ from those in the study by Walkey et al^10^ and Lang et al^12^ but corroborate more recent studies.^11,46,47^ The segmented regression approach in the study by Walkey et al^10^ does not involve the comparison of treated and control units and, as such, cannot account for factors external to the time series.^48,49^ Lang et al^12^ did not match the covariates in deriving the synthetic control. The emerging literature suggests that a good balance in covariates requires lenient assumptions and provides synthetic control estimators with tighter bounds.^27,50^ Unlike past work, which has focused on a single state, we focused on multiple states and used novel and sophisticated statistical methods. DiD analysis controlled for demographic characteristics that could affect individuals’ vaccination decisions.^51^

### Limitations

This study has limitations. Although creating an ideal control group is the key idea of synthetic control methods, there are some practical difficulties in creating one. The pretreatment trends of New Mexico, West Virginia, and Washington were somehow different from their synthetic counterparts. We advise caution in making inferences about the results for these states. Moreover, our analysis is subject to possible data collection and reporting issues of statewide daily vaccinations and the potential self-reporting bias in the HPS responses.

## Conclusions

In this study, vaccine lottery incentives were associated with increased vaccinations overall and in Ohio, Maryland, Oregon, and Washington but not in Arkansas, Kentucky, and West Virginia. This heterogeneity in results should inform governments when adopting similar policies in the future. The findings may also be relevant to the ongoing debate on how to persuade the millions of US residents who are not yet vaccinated against COVID-19.

## Notes

Supplement. **eTable 1. **Announcement Dates of Lottery-Incentive Programs for COVID-19 Vaccinations in the United States **eFigure 1. **Daily COVID-19 Vaccination Rate per 100 000 People in the United States, 2021 **eFigure 2. **Event Study Graphs From DiD Analysis **eTable 2. **Summary Statistics of State-Level Variables in Treated and Control States **eTable 3. **Weights of Control States Used in Calculating Synthetic Treated States in Ridge-Augmented Synthetic Control Analysis **eFigure 3. **Trends of Daily Vaccination Rate in Treated States and Their Synthetic Controls **eFigure 4. **Gap Plots From the Augmented Synthetic Control Analysis **eFigure 5. **Sensitivity Analysis Gap Plot From Staggered Synthetic Control Method **eFigure 6. **Observed and Synthetic Trend Plots From Sensitivity Analysis **eFigure 7. **Gap Plots From Sensitivity Analysis
