# Supplementary material for: Implementation of State Vaccine Incentive Lottery Programs and Uptake of COVID-19 Vaccinations in the United States
Source: JAMA Netw Open. 2021 Dec 9;4(12):e2138238. doi: 10.1001/jamanetworkopen.2021.38238 (PMC8662369; doi:10.1001/jamanetworkopen.2021.38238)
Supplement: Supplement. — eTable 1. Announcement Dates of Lottery-Incentive Programs for COVID-19 Vaccinations in the United States eFigure 1. Daily COVID-19 Vaccination Rate per 100 000 People in the United States, 2021 eFigure 2. Event Study Graphs From DiD Analysis eTable 2. Summary Statistics of State-Level Variables in Treated and Control States eTable 3. Weights of Control States Used in Calculating Synthetic Treated States in Ridge-Augmented Synthetic Control Analysis eFigure 3. Trends of Daily Vaccination Rate in Treated States and Their Synthetic Controls eFigure 4. Gap Plots From the Augmented Synthetic Control Analysis eFigure 5. Sensitivity Analysis Gap Plot From Staggered Synthetic Control Method eFigure 6. Observed and Synthetic Trend Plots From Sensitivity Analysis eFigure 7. Gap Plots From Sensitivity Analysis [file jamanetwopen-e2138238-s001.pdf]

Supplemental Online Content

Acharya B, Dhakal C. Implementation of state vaccine incentive lottery programs and uptake of COVID-19 vaccinations in the United States. *JAMA Netw Open.* 2021;4(12):e2138238. doi:10.1001/jamanetworkopen.2021.38238

eTable 1. Announcement Dates of Lottery-Incentive Programs for COVID-19 Vaccinations in the United States

eFigure 1. Daily COVID-19 Vaccination Rate per 100 000 People in the United States, 2021

eFigure 2. Event Study Graphs From DiD Analysis

eTable 2. Summary Statistics of State-Level Variables in Treated and Control States

eTable 3. Weights of Control States Used in Calculating Synthetic Treated States in Ridge-Augmented Synthetic Control Analysis

eFigure 3. Trends of Daily Vaccination Rate in Treated States and Their Synthetic Controls

eFigure 4. Gap Plots From the Augmented Synthetic Control Analysis

eFigure 5. Sensitivity Analysis Gap Plot From Staggered Synthetic Control Method

eFigure 6. Observed and Synthetic Trend Plots From Sensitivity Analysis

eFigure 7. Gap Plots From Sensitivity Analysis

This supplemental material has been provided by the authors to give readers additional information about their work.

© 2021 Acharya B et al. *JAMA Network Open.*

eTable 1. Announcement Dates of Lottery-Incentive Programs for COVID-19 Vaccinations in the United States

State	Announcement Date	Included in Analysis	If excluded in analysis, reasons(s)
Arkansas	5/26/21	Yes	
California	5/27/21	Yes	
Colorado	5/25/21	Yes	
Delaware	5/25/21	No	State has implemented smaller prizes and other programs
Kentucky	6/4/21	Yes	
Louisiana	6/17/21	No	Announcement after study window
Massachusetts	6/15/21	No	Announcement after study window
Maine	6/16/21	No	Announcement after study window
Maryland	5/20/21	Yes	
Michigan	7/1/21	No	Announcement after study window
North Carolina	6/10/21	No	Announcement after study window
New York	5/20/21	Yes	
New Mexico	6/1/21	Yes	
Nevada	6/17/21	No	Announcement after study window
Ohio	5/13/21	Yes	
Oregon	5/21/21	Yes	
Washington	6/3/21	Yes	
West Virginia	6/1/21	Yes	

© 2021 Acharya B et al. *JAMA Network Open.*

eFigure 1. Daily COVID-19 Vaccination Rate per 100 000 People in the United States, 2021

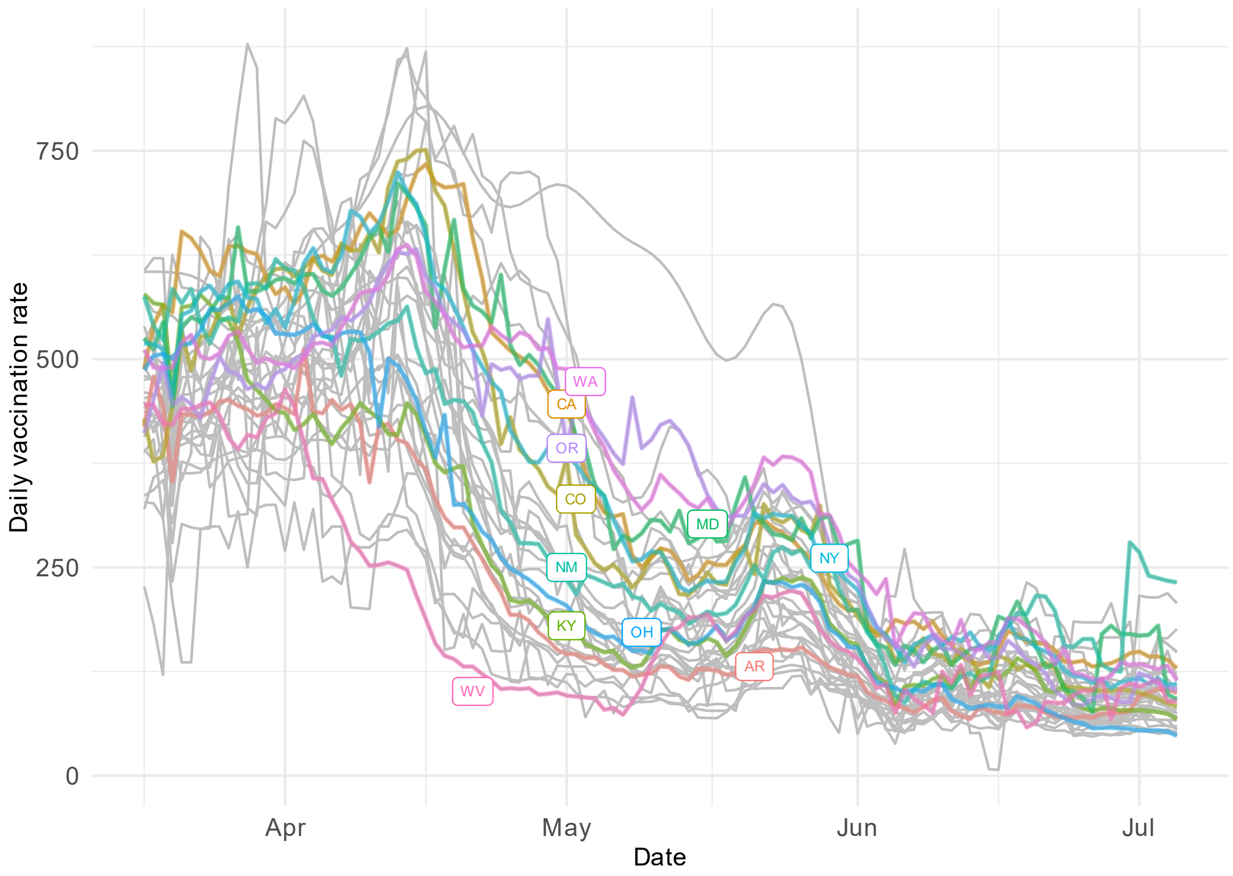

© 2021 Acharya B et al. *JAMA Network Open.*

eFigure 2. Event Study Graphs From DiD Analysis

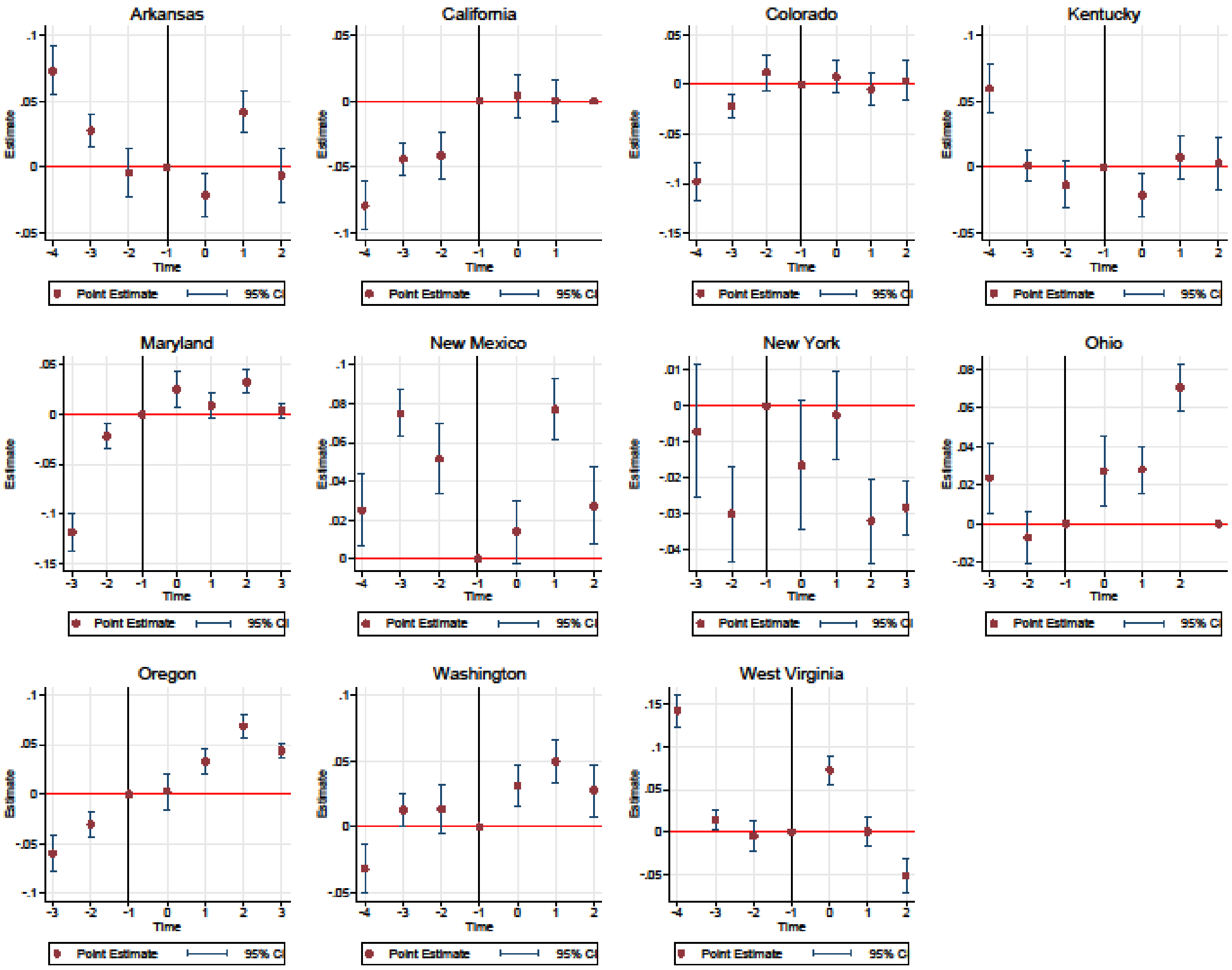

© 2021 Acharya B et al. *JAMA Network Open.*

eTable 2. Summary Statistics of State-Level Variables in Treated and Control States

Characteristics	Treated states<br>Mean (Standard deviation)	Control states<br>Mean (Standard deviation)
No. of states	11	28
Mean daily vaccination rate per 100,000	312.1 (183.7)	273.7 (190.0)
% Black	9.46 (8.58)	11.93 (11.89)
% Hispanic	16.71 (15.2)	10.87 (8.84)
% Population aged 65 years or more	16.96 (1.67)	16.7 (2.1)
% Population aged 14 years or less	18.05 (0.85)	18.74 (1.94)
% Unemployment rate	4.15 (0.53)	3.44 (0.78)
% Education at least college degree	32.22 (7.31)	32.53 (6.73)
% Population in labor force	62.11 (4.44)	64.53 (3.75)
% With private health insurance	65.78 (5.88)	69.81(5.19)
Annual median household income (dollars)	65712(14089)	63592 (9372)
Party lean index	0.08 (22.88)	-12.05 (24.09)
Urban index	10.76 (1.15)	10.25 (1.14)
Life expectancy at birth	78.07 (2.31)	78.14 (1.59)

© 2021 Acharya B et al. *JAMA Network Open.*

eTable 3. Weights of Control States Used in Calculating Synthetic Treated States in Ridge-Augmented Synthetic Control Analysis

Donor pool of control states	Treated states										
	AK	CA	CO	KY	MD	NM	NY	OH	OR	WA	WV
AZ	0.031	0.67	0.17	-0.237	-0.009	0.303	-0.002	0	0.549	0.249	0.333
CT	-0.03	-0.111	0.15	-0.042	0.073	0.04	0.001	-0.001	-0.041	0.002	0.031
DC	0.147	0.178	0.221	-0.308	0.273	-0.081	-0.002	0.003	0.024	0.002	-0.223
FL	0.653	0.018	0.097	0.052	-0.049	0.026	-0.002	-0.001	-0.03	-0.001	-0.164
GA	-0.083	0.19	-0.047	0.261	0.112	-0.045	-0.001	0	-0.155	-0.007	-0.208
ID	0.146	0.453	0.323	-0.654	-0.094	0.112	0.083	-0.001	0.18	0.004	-0.933
IN	0.201	0.018	-0.138	-0.182	-0.097	-0.112	0	0.197	0.006	-0.003	-0.083
IA	-0.131	0.104	0.057	-0.263	-0.073	0.389	0.001	-0.002	0.091	0	0.146
KS	0.304	0.125	0.144	0.059	-0.108	0.292	0.002	0.375	-0.054	-0.002	-0.277
MN	0.048	-0.027	-0.007	-0.081	-0.013	-0.341	0.208	0	0.073	0.296	0.068
MS	0.013	-0.135	-0.138	0.14	-0.039	-0.217	-0.002	0.107	0.032	-0.002	-0.221
MO	0.082	0.007	-0.103	0.093	0	0.111	-0.002	0	0.001	-0.003	-0.383
MT	0.312	0.08	0.017	1.022	0.036	-0.382	0	-0.003	0.162	0.002	0.186
NE	0.14	-0.06	-0.033	0.072	0.093	0.302	0	0	-0.094	-0.002	0.819
ND	-0.085	-0.117	0.061	0.299	-0.077	-0.193	-0.002	0.001	0.096	0.001	0.388
PA	-0.015	-0.062	-0.22	0.382	0.043	0.012	0.001	0.002	-0.131	0	-0.18
RI	-0.151	-0.031	-0.08	0.128	0.116	-0.046	0	-0.002	-0.048	0.003	0.066
SC	-0.291	-0.244	-0.029	0.239	0.094	-0.066	-0.001	0.019	-0.06	-0.004	0.369
SD	-0.3	-0.213	-0.126	-0.248	0.013	-0.09	0.002	0.001	-0.093	-0.003	0.041
TN	0.027	-0.453	0.036	0.025	0.078	0.012	-0.001	0.103	-0.031	-0.003	0.748
TX	0.202	0.309	0.107	0.427	-0.036	0.286	-0.001	-0.004	-0.124	0.003	0.163
UT	0.013	-0.17	0.08	-0.128	0.067	-0.061	0.117	-0.002	-0.022	0.001	-0.133
VT	-0.183	-0.168	0.033	-0.453	-0.196	-0.047	0.001	0.206	0.069	0.001	0.073
VA	-0.248	0.264	0.108	0.237	0.352	0.272	0.283	0	0.589	0.466	0.266
WI	0.102	0.462	0.234	0.25	0.437	0.53	0.317	0.002	-0.062	0	-0.003
WY	0.095	-0.083	0.083	-0.09	0.005	-0.005	-0.001	0	0.073	0.002	0.11

© 2021 Acharya B et al. *JAMA Network Open.*

eFigure 3. Trends of Daily Vaccination Rate in Treated States and Their Synthetic Controls

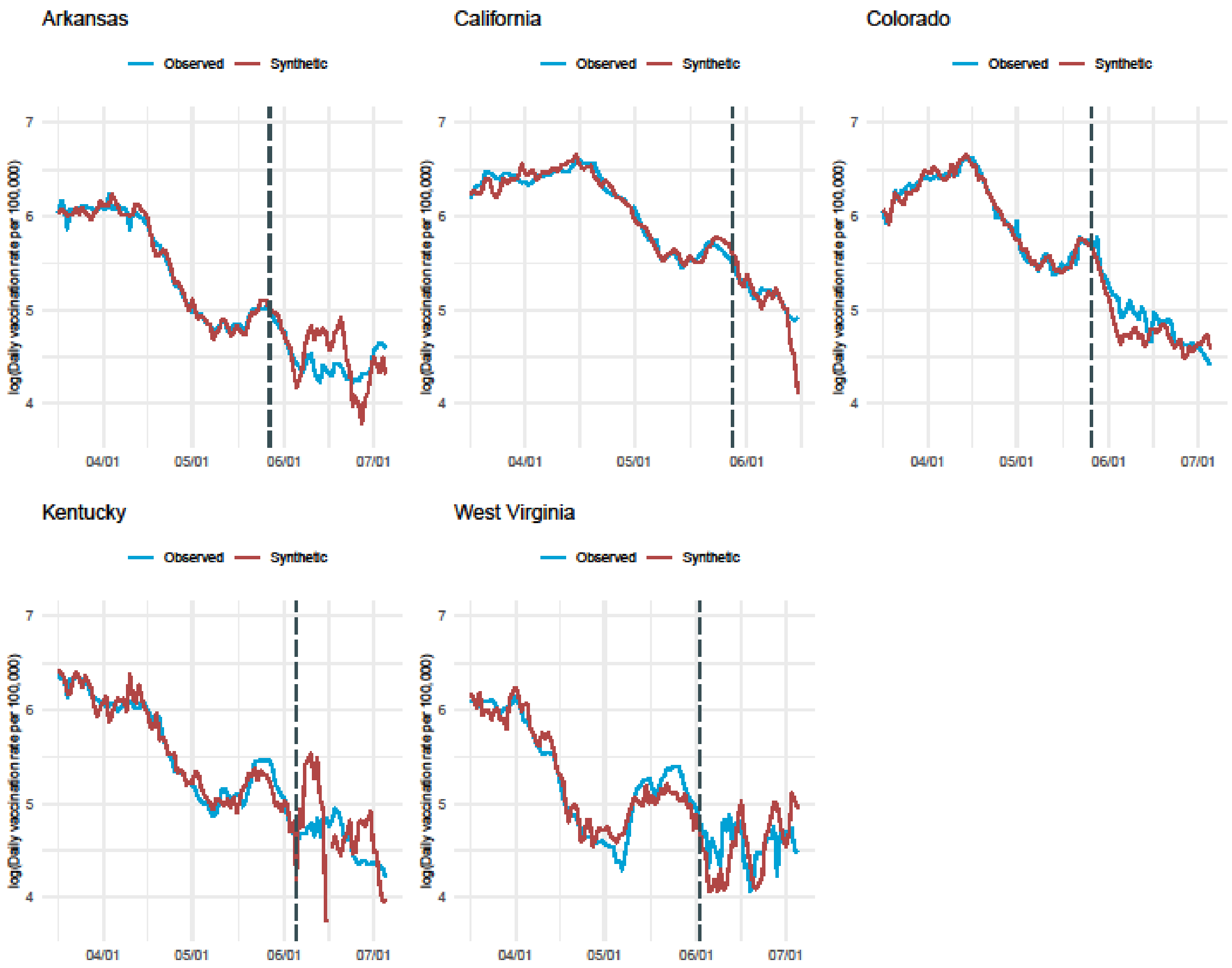

© 2021 Acharya B et al. *JAMA Network Open.*

eFigure 4. Gap Plots From the Augmented Synthetic Control Analysis

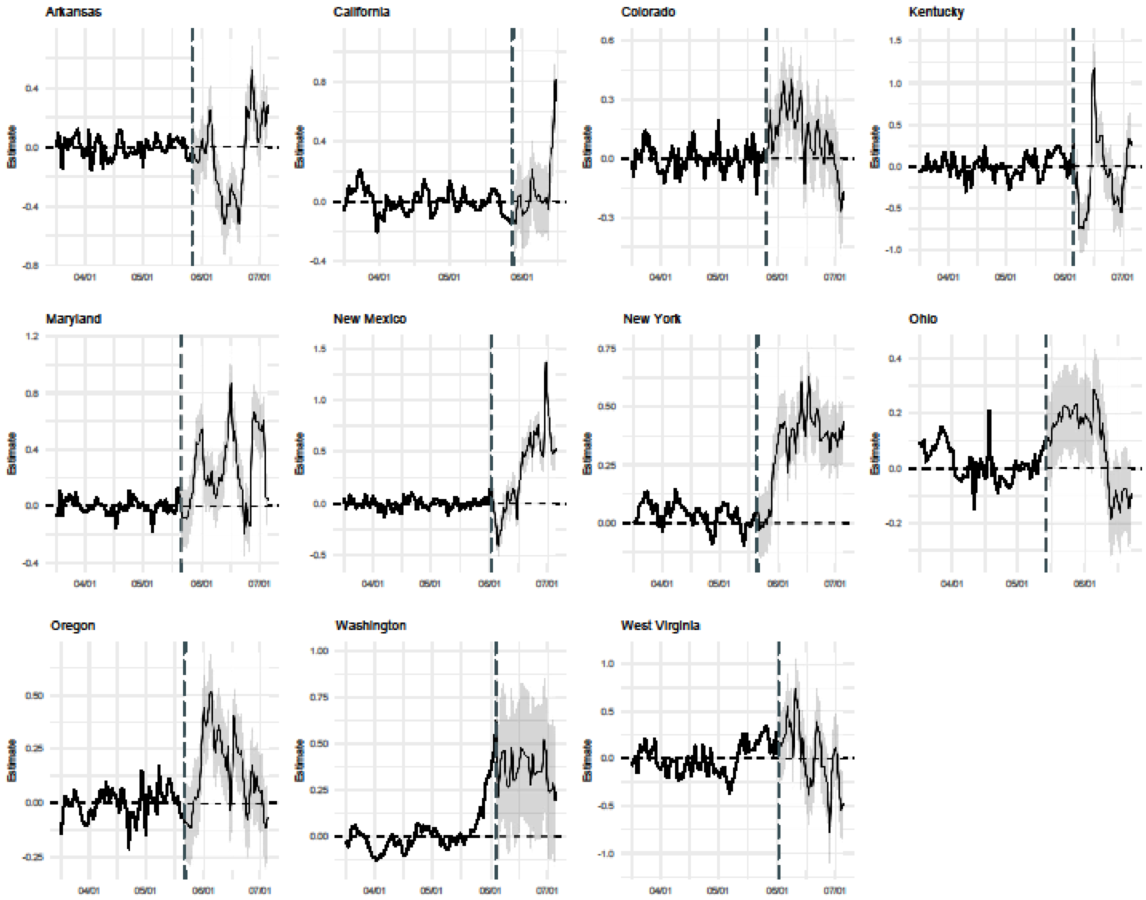

© 2021 Acharya B et al. *JAMA Network Open.*

eFigure 5. Sensitivity Analysis Gap Plot From Staggered Synthetic Control Method

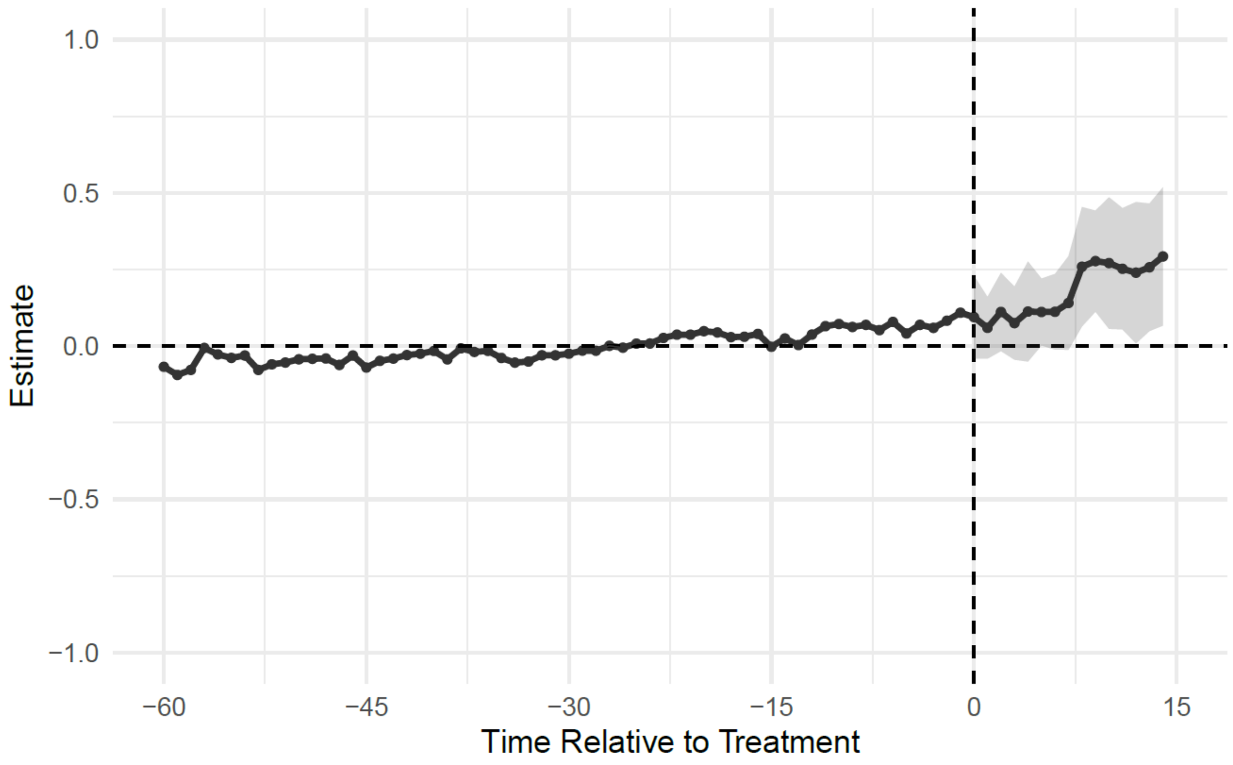

© 2021 Acharya B et al. *JAMA Network Open.*

eFigure 6. Observed and Synthetic Trend Plots From Sensitivity Analysis

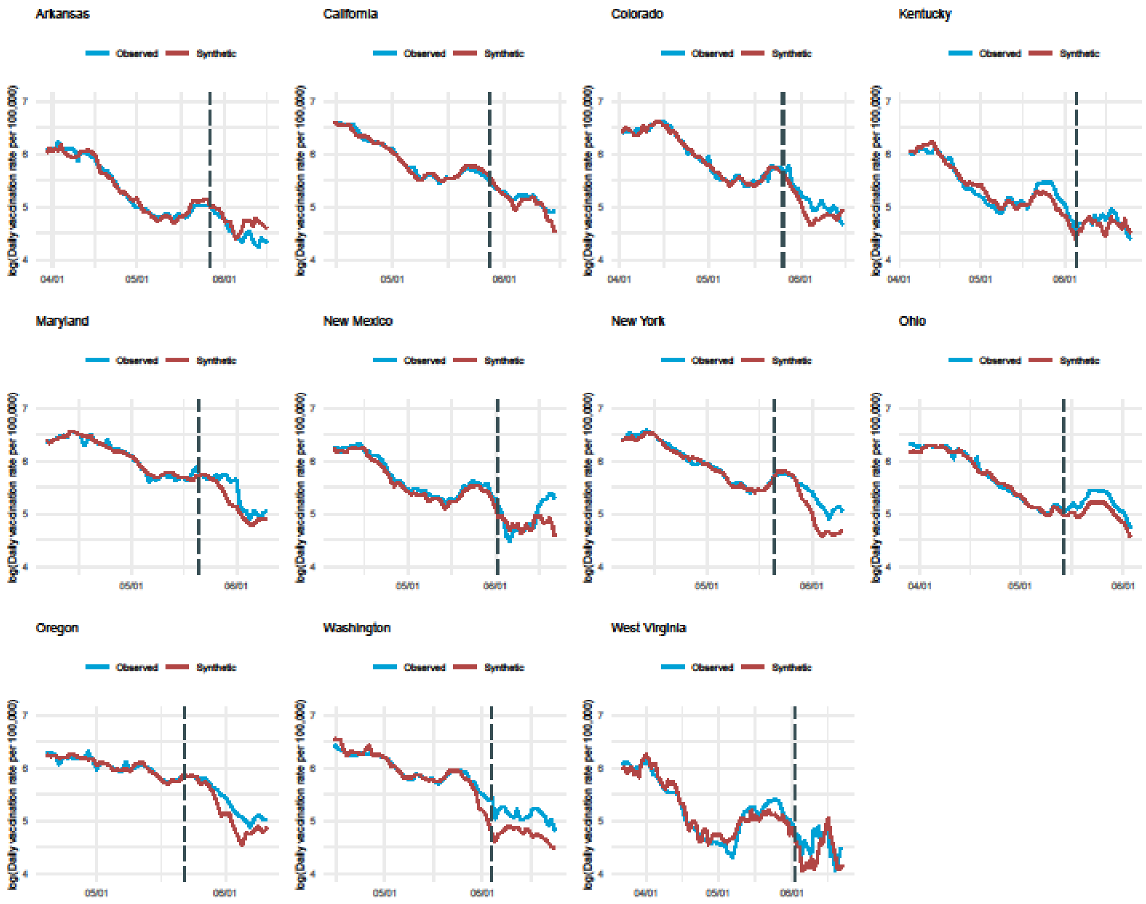

© 2021 Acharya B et al. *JAMA Network Open.*

eFigure 7. Gap Plots From Sensitivity Analysis

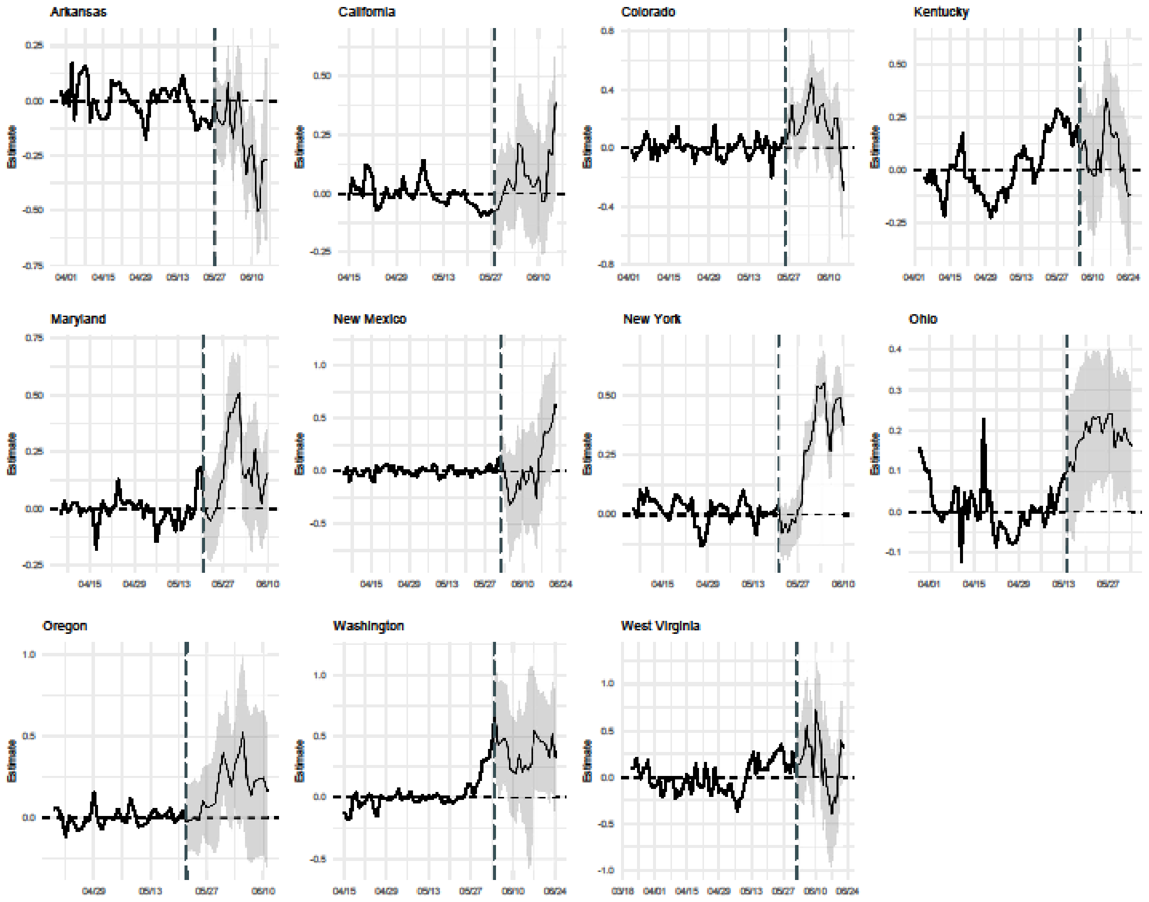

© 2021 Acharya B et al. *JAMA Network Open.*
